# Challenges and Alternatives to Plastics Recycling in the Automotive Sector

**DOI:** 10.3390/ma7085883

**Published:** 2014-08-15

**Authors:** Lindsay Miller, Katie Soulliere, Susan Sawyer-Beaulieu, Simon Tseng, Edwin Tam

**Affiliations:** Environmental Engineering, University of Windsor, 401 Sunset, Windsor, ON N9B 3P4, Canada; E-Mails: soulliv@uwindsor.ca (K.S.); susansb@uwindsor.ca (S.S.-B.); tsengc@uwindsor.ca (S.T.); edwintam@uwindsor.ca (E.T.)

**Keywords:** end-of-life vehicles, plastics recycling, waste management, automobile, energy recovery, light-weighting, renewable plastics, ecodesign, shredder residue, environmental impact

## Abstract

Plastics are increasingly a preferred material choice in designing and developing complex, consumer products, such as automobiles, because they are mouldable, lightweight, and are often perceived to be highly recyclable materials. However, actually recycling the heterogeneous plastics used in such durable items is challenging, and presents very different scenarios to how simple products, such as water bottles, are recovered via curbside or container recycling initiatives. While the technology exists to recycle plastics, their feasibility to do so from high level consumer or industrial applications is bounded by technological and economical restraints. Obstacles include the lack of market for recyclates, and the lack of cost efficient recovery infrastructures or processes. Furthermore, there is a knowledge gap between manufacturers, consumers, and end-of-life facility operators. For these reasons, end-of-life plastics are more likely to end up down-cycled, or as shredder residue and then landfilled. This paper reviews these challenges and several alternatives to recycling plastics in order to broaden the mindset surrounding plastics recycling to improve their sustainability. The paper focuses on the automotive sector for examples, but discussion can be applied to a wide range of plastic components from similarly complex products.

## 1. Introduction

The use of lightweight plastics and composite materials in the automotive industry has been increasing in recent years due to legislative and consumer demands for lighter weight, fuel-efficient vehicles. The use of these materials has been credited with lowering the average vehicle weight by 200 kg [1]. In some cases, plastics are replacing heavier ferrous materials whereas, in other cases, plastics and composites are being added for consumer comfort purposes. In addition to being lightweight, these materials are also durable and easily molded. Substituting heavier materials with plastics leads to an overall weight reduction, with a 10% weight reduction resulting in a 3% to 7% improvement in fuel efficiency [2]. However, the increasing use of plastics shifts the environmental burden from the use phase of an automobile (emissions reduction) to the end-of-life vehicle (ELV) stage (materials disposal). Whether light-weighting results in an overall reduction in environmental impacts, may be soon a debatable question. A previous study concluded lighter weight vehicles show improved environmental performance even with 100% landfilling of plastic parts [3]. However, another study concluded that the environmental benefit of light-weighting would break even with negative ELV environmental impacts after approximately 132,000 km of vehicle travel [4]. Even with the use phase emissions reductions, it is critical to understand and address the ELV scenario if the use of plastics in the automotive sector can be truly considered as a sustainable option.

Plastics and composites recycling in the automotive industry is complex and challenging. Although simple plastic products (e.g., water bottles, food containers) are readily recyclable, plastics and composites in automotive applications are heterogeneous, have strong connections to other plastics, and are thus difficult to liberate for recycling [5]. Thermoset materials present a further challenge since they cannot be melted down and recycled due to their permanent cross-link structure. Even when a material can be recycled, it is often still landfilled because it cannot actually be physically recovered [5]. The placement of foam, for example, is typically in an area of a vehicle where it cannot be readily accessed and then separated from other materials: it will eventually be contaminated with other materials (e.g., fluids). Furthermore, there are additional obstacles blocking the recycling routes such as a lack of technology and market for recyclates. Lastly, next generation materials, such as carbon fiber, further complicate recycling due to their inherent complexity [6]. It is generally found along with other materials, and is difficult to separate. Bio-based plastics present an interesting alternative to petroleum plastics but the technology needs maturity before implementation.

**Figure 1:**
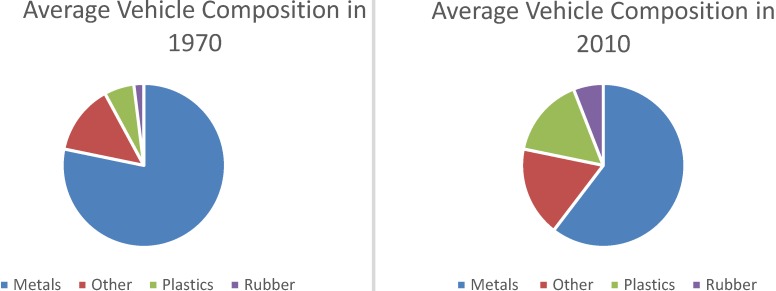
Change in vehicle composition from 1970 to 2010 [8].

For a spent automobile, the current ELV recovery and recycling process consists of dismantling, de-pollution and shredding, physical and mechanical treatment of shredder residue (SR), and treatment of SR by energy recovery [7]. Presently, plastics and composites contribute to SR, and as the use of these materials increases, so does the amount of SR generated. The percent plastics by mass in an average vehicle has gone from 6% in 1970 up to 16% in 2010 and is expected to reach 18% in 2020 (see Figure 1) [8]. The automotive industry accounts for a significant percentage of plastics demand with estimates ranging up to 30% and on the rise [9,10].

Identifying possible improvements and alternatives in the plastics recycling chain is of utmost importance in Europe given upcoming legislation that will require 95% of a vehicle to be processed for “reuse and recovery” and 85% be processed for “reuse and recycling” by 1st January 2015 [11]. This legislation limits the amount of the ELV that can be dealt with through recovery and encourages recycling whenever environmentally viable. It is expected that by increasing the recycling of plastics from SR, an additional 6%–10% of the entire ELV mass can be recycled, therefore making this a key initiative towards meeting legislative requirements [12]. While North America does not have similar legislation, the global nature of the automotive industry has resulted in many manufacturers adopting global initiatives towards regional environmental requirements.

In summary, the recycling of plastics and composites from complex, durable products is limited by technological and economical restraints. The challenges in recycling plastics and composites from ELVs stem from a lack of market for recyclates, a lack of infrastructure, economics, knowledge gap, and heterogeneous mixes of plastics. This paper examines the challenges of plastics recycling in the North American automotive industry and suggests the following alternatives to overcome these challenges: (1) pursue plastics reuse and refurbishment; (2) pursue recycling; (3) pursue recovery without segregation (energy recovery); (4) move to renewable plastics; and (5) move away from plastics.

## 2. Challenges and Obstacles Associated with Plastics Recycling

**Figure 2:**
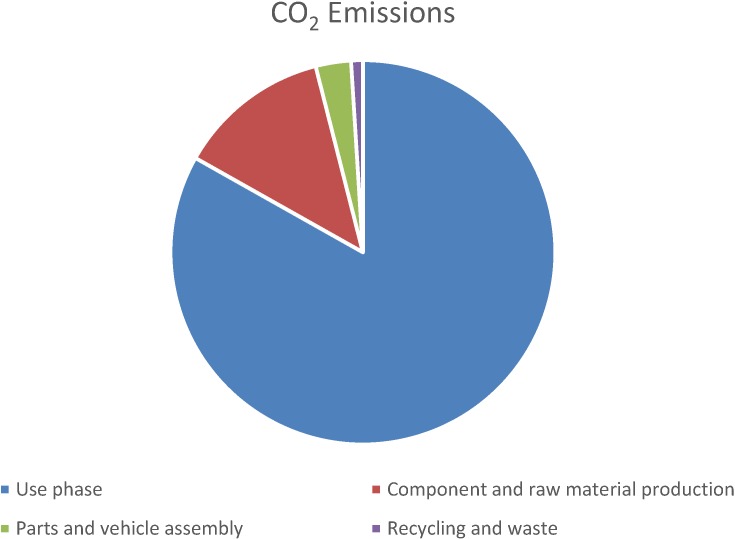
CO_2_ emissions during various life stages of an average vehicle (1000 kg) [8].

In order to meet the requirements for CO_2_ emission reductions, vehicles are being light weighted with more plastics substituting for heavier materials, such as steel, where possible. CO_2_ emissions originate predominantly from the use phase, with only ~1% being attributed to the recycling and waste stage (see Figure 2) [8]. In this regard, plastics are being credited for lessening environmental impacts. However, only a few plastic parts actually are recycled; typically these are the fascia (or “bumpers”), dashboards, and battery casings [1].

An important distinction to make in the discussion of difficulties surrounding plastics recycling is that between thermosets and thermoplastics. Thermoset materials cure into a given shape through the application of heat. Curing results in permanent cross-links resulting in a high degree of rigidity, however, changes the material permanently. Thermoset materials will not remelt or regain processability. The only option for mechanical recycling is to pulverize these materials for reuse as fillers [13]. In contrast, thermoplastic materials become pliable when heated, allowing them to be moulded, but they do not set. These materials typically begin in pellet form and are heated and moulded. As the material cools it will harden, but no curing takes place and cross-links are not formed. This allows for thermoplastic materials to be reprocessed many times although continual recycling will result in degradation. Although there are more options for recycling of thermoplastics, thermosets can be used for other applications such as in the case of polyurethane foam (PU) foam which is commonly shredded and used as carpet underlay.

There are several obstacles to recycling the types of materials that are commonly used in automotive applications. Plastics that are commonly used in automotive applications include: polyurethane foam (PU) polypropylene (PP), polyethylene terephthalate (PET), polyethylene (PE), polyvinyl chloride (PVC), and acrylonitrile butadiene styrene (ABS). Polyurethane foam, for example, has been gaining widespread use in the automotive industry due to its lightweight, mouldable, and durable properties. It is also a useful noise dampener. These desirable properties are responsible for the increase in usage of this material within vehicles despite it being a thermoset material and other materials being more easily recycled [14]. Polyurethane is commonly found in automotive seating applications as well as within the interior and under the hood. Unlike some other thermoset materials, polyurethane has the advantage of being relatively easy to convert back into its original monomer [15]. Therefore, this, in theory, is recyclable and several technologies exist for this purpose. In reality, the location of the foam within the vehicle is not easily accessible and is often contaminated. Furthermore, it is currently not economically viable for dismantlers to segregate this inexpensive material from the end of life vehicles. Some other vehicle components that are commonly made of plastics include drink trays (PP), armrest finishers (PP/ABS), and seatbelts (PET). Similar to PU foam components, these often do not make it to recycling due to inaccessibility and lack of economic incentive at the dismantling stage.

Carbon fibre reinforced plastic is another composite that is theoretically recyclable yet is often landfilled. This material is also used in the automotive industry although usually reserved for high end vehicles and racing cars due to its higher costs. Recycling carbon fibre reinforced plastic is inherently difficult due to its complex composition and cross-linked nature [16]. Additionally, it is usually found in combination with other materials such as metal fixings and hybrid composites, and as a result, it is difficult to separate them for subsequent recycling.

PLA is a renewable bio-plastic made from the fermentation of starches utilized in automotive interior components such as dashboard trim and spare tire cover. Current methods for PLA recycling are mechanical and chemical reprocessing, and unlike conventional plastics, composting. Mechanical processing has shown to reduce the physical properties at each application [17]. For mechanical processing, a homogeneous (sorted) feedstock is necessary to preserve the physical properties. For composting, PLA is a biodegradable plastic under industrial composting conditions according to EN 13423, ASTM D6400, and D6868. The challenges associated with PLA recycling are identifying and then sorting them. With respect to infrastructure, the challenges are cost and implementation. Similar to other commodity plastics, two-thirds of the total financial cost in plastic recycling is incurred by collection and sorting [18].

Several common obstacles can be established based on the discussion to date.

### 2.1. A Lack of Market for Recyclates

The benefit of segregating and targeting a material for recycling is diminished if there is no market for the material once it is recycled. The cost of some of these materials is already low; for example, polyurethane foam. Furthermore, even for meltable thermoplastics the mechanical properties of these materials may be altered during the recycling process, rendering them less suitable to be recycled for their original purpose. Materials used within the vehicle are also often times contaminated, such as PU foam used as an engine compartment would likely become contaminated with oils. Similarly, carbon fibre undergoes physical changes as a result of the recycling process preventing its reintroduction as a direct substitute for virgin fibres. For these reasons, these materials are more likely to be down-cycled than truly recycled, especially in the case of thermoset materials. PU foam for example, has been down-cycled for use in carpet underlay whereas carbon fibres have been in the construction industry as fillers for artificial woods and asphalt [19]. The use of foams and composites is increasing; however, the market size for these rebounded applications is not large enough to accompany the recycled materials.

### 2.2. A Lack of Infrastructure

Plastic manufacturers recycle their own scrap materials in house. Once the plastics leave the manufacturers for use in their end application, such as automotive components, the recycling process is complicated by a lack of infrastructure. Current automobile recycling infrastructure consists of a dismantler, shredder, and non-ferrous operator. The dismantler, after the de-pollution step, removes components with sufficient market value such as aluminum rims and catalytic converters, for reuse, remanufacturing or recycling. Parts recovery values in the industry will vary depending on the condition, makes, models, and ages of the ELVs processed, as well as the market demand for the particular part and assembly types. In general, this can amount to as little as 5% to as much as 42% of the vehicle mass [7,20]. The remainder is sent to a shredder facility. Post-shredding, mechanical and magnetic separation processes allow for the recovery of ferrous metals.

Approximately 15%–25% of the mass is leftover and ends up at a landfill in the form of shredder residue (SR). Foams, plastics, and polymer composites typically end up as SR. Upcoming European legislation will force a reduction to 5%, which will require further segregation and recycling of the plastic materials. In order to target these materials, a change in infrastructure will be required. In addition to meeting more stringent regulations is the concern over the changes in SR composition. It is expected that new vehicle composition will reach 10%–15% plastic materials compared to the current 6%–8% [21].

### 2.3. Economics

The previous two obstacles are both largely based on economics. Without a sound market for recyclate, it is not economical to recycle these materials. Dismantling operations are based on recovering materials for profit, and cannot afford to separate low values materials. Furthermore, it is not economical to improve or develop infrastructure for new technologies if no future revenue is expected to be generated. In the absence of a real market for the reuse or recycle of these materials, the economic situation will likely only be addressed through legislative efforts. One exception are carbon fibre materials, which have high manufacturing and material costs as well as disposal costs and could benefit economically from recycling practices.

### 2.4. Mindset and Knowledge Gap

The mindset of the public is that plastic materials are easily recycled based on the public knowledge of recyclable products such as water bottles, which are typically made from a single thermoplastic material type, not bonded to any other items, and are easily collected and transported. Manufacturers and consumers also favour plastics to reduce the overall vehicle weight and thereby lower fuel consumption and subsequent emissions. Although plastics are lighter weight than some ferrous materials, replacing parts with plastics will not always yield a positive environmental impact over the life of a vehicle. This poses a knowledge gap as manufacturers may select a plastic component partially based on its ability to be recycled. This was demonstrated using a case study of material selection for an engine cover [22]. Although PU foam was selected for its recyclability, the end-of-life circumstances were such that no matter what material was selected for this part, it would be landfilled. In this case, the best material selection would have been the lightest weight component to reduce the use phase environmental impacts. However, as plastic usage continues to rise, there will likely be consequences at the end-of-life stage, such as a percentage increase in landfilling, if sustainable solutions cannot be achieved.

### 2.5. Heterogeneous Materials and Heterogeneous Mixture within an Application

The heterogeneous nature of these materials makes it difficult to recover without losing some of their original mechanical properties. Moreover, in addition to the heterogeneity of these materials, they are often coated. Coating materials can compromise the properties of recycled plastics [23]. Perhaps even more difficult is the heterogeneous mixture of materials within an application. For example, within the seating section of a vehicle, there can be five different types of plastics and composites, some being thermoplastics and others thermosets. This complicates the recovery process since in order for it to be effective, all of these materials would have to be recovered and separated at the end of life processing. This increases the time required to dismantle a vehicle and could reduce the cost effectiveness of the process if commercial materials recyclers having suitable materials separation technologies are not readily available. Another option is to separate materials post shredding; however, unlike steel and light metals, plastics are not easily separated from one another due to their overlapping physical properties [24].

As a result of these obstacles, what is *technically possible* in terms of recycling are often times not *practically feasible*. The reality is that most often the plastics and composites used within the automotive sector end up as automotive shredder residue and have consequently been landfilled. With landfilling no longer an option in Europe, there is a pressing need to develop feasible solutions.

## 3. Alternative Solutions for Plastics Recycling

### 3.1. Alternative 1—Pursue Plastics Reuse/Refurbishment

According to Sawyer-Beaulieu’s 2009 study, as much as 12% on average by weight of end-of-life vehicles (ELVs) entering the dismantling processes are recovered and directed for either, reuse, remanufacturing or recycling, including the recovered fluids. Of this 12%, more than 80% of these recovered end-of-life materials and components (almost 10% by weight of the processed ELVs) are parts and parts assemblies directed for reuse, remanufacturing and recycling. Parts and assemblies directed for reuse and remanufacturing were almost 6% weight of the ELVs processed and represented hundreds of different part and assembly types [20].

The materials compositions of these reusable and remanufacturable parts and assemblies vary in complexity. These include: (1) parts composed principally of metals (e.g., engines, transmissions, AC compressors, steering gears, radiator supports, alternators, starters, *etc.*) and very amenable to reuse and/or remanufacturing (or refurbishment); and (2) parts assemblies of relatively complex materials compositions, having significant non-metallic materials content (refer to examples in Table 1, below). Should these latter types of parts be resold for reuse, then these represent instances of plastics reuse even if it is incidental reuse of the onboard plastics. Based on literature available in the public domain, the environmental benefits of “incidental” plastics reuse, through the reuse of automotive parts or assemblies having complex materials compositions, have not been explored.

**Table 1:** Examples of parts assemblies having significant non-metallic materials content [20].

Part Assembly	Average		Range	
	Metals (% Weight of part)	Non-Metals (% Weight of part)	Metals (% Weight of part)	Non-Metals (% Weight of part)
Front door assembly	70%	30%	66%–72%	28%–34%
Rear door assembly	69%	31%	66%–74%	26%–34%
Steering wheel	60%	40%	55%–65%	35%–45%
Steering column	80%	20%	77%–83%	17%–23%
Dash assembly	44%	56%	28%–54%	46%–72%
Seat assembly	66%	34%	21%–81%	19%–79%

Fascia, or commonly the front and rear “bumper covers”, are one of the few large, predominantly plastic automotive parts that are readily recovered by dismantlers and sold for direct reuse and refurbishment and reuse [20]. There are companies in Canada and the U.S. that refurbish and sell recovered bumper covers as alternatives to using new original equipment manufacturer (OEM) or aftermarket bumper covers. LKQ Corporation/Keystone Automotive, for example, own approximately 37 facilities dedicated to bumper cover repair in the United States, Canada and Mexico that employ approximately 600 workers [25]. Fascia refurbishment is one of the limited examples of direct reuse—or refurbishment and reuse—of automotive parts that are made principally or exclusively of plastic, likely because they are readily identifiable, isolated, and relatively easy to handle. Nevertheless, the recovery and subsequent resale of other similar plastic assemblies from automobiles or other consumer products merits serious consideration as part of the larger effort to achieve materials sustainability.

### 3.2. Alternative 2—Pursue Recycling

Technologically, recycling is possible for the plastic and composite materials used in the automotive industry. Excellent reviews of the technical aspects and options for recycling plastics and composites are available [1,15,16,26]. While recycling is practically limited by the above mentioned obstacles, it may still be a preferred option for these materials and can help to alleviate the amount of materials being landfilled if some of the obstacles can be overcome. The feasibility of recycling can be improved through a few major avenues.

#### 3.2.1. Improve the Quality of the Recyclate

If recycling these materials is to succeed, plastic and composite waste need to be rendered into a valuable resource. This will help drive higher value applications for recyclate. In order to move in this direction, the by-products resulting from mechanical recycling processes would need to have similar properties to commercial grade plastics with respect to their type and monomer origin [26].

New innovative technologies will be paramount to improving recyclate quality. One such technology is Polyfloat^®^, developed by SiCon, which enables high-precision density separation of plastics from shredder residue. The process relies on a lamella separation system and is applicable to the plastics that are commonly found within vehicles [27]. Another option would be to find more uses for SR, in the event dismantling more material before shredding proves to be uneconomical [7].

The value of the recyclate derived from thermoset materials can be improved by finding applications in which the properties of this recyclate can by uniquely applied. For example, using the recyclate as a permeable core that allows it to act as a flow layer [28] as well as using it to provide damping in a composite for the purpose of noise insulation [29]. Further improvements in thermoset recycling lie in the development of new materials that have the desirable properties of a thermoset material however are recyclable at the ELV stage. Advancements have been made recently towards this improvement [30].

#### 3.2.2. Establish Industry Partnerships

The most likely route to achieving recycling success is through developing mutually beneficial partnerships where recycling benefits all stakeholders. Because recycling these materials often does not result in the direct substitution of virgin material for the part’s original purpose, the next preferred solution is to find a suitable purpose for the recycled material. By establishing collaborations between waste generators and recyclers, the generators can focus on material sorting and reduction of contamination to reduce the processing burden on the recycler. An example of this is the collaboration between Boeing and BMW. With Boeing’s 787 Dreamliner made of 50% carbon fibre material, recycling it at end of life is essential. BMW is working to bring two vehicles with a carbon passenger cell onto the market and therefore has a potential use for the recycled carbon fibres. In a joint venture between the two companies, infrastructure has been developed to turn the carbon fibre material into fabric, which is then processed to make body components for BMW [31]. This partnership, while nascent, serves as an example of industrial ecology to encourage other companies to seek out mutually beneficial collaborations. Since carbon fibre is also used in several sectors such as automotive, aircraft, and watercraft, recycling partnerships could be easily established if infrastructure were available.

#### 3.2.3. Incentives and Legislation

**Figure 3:**
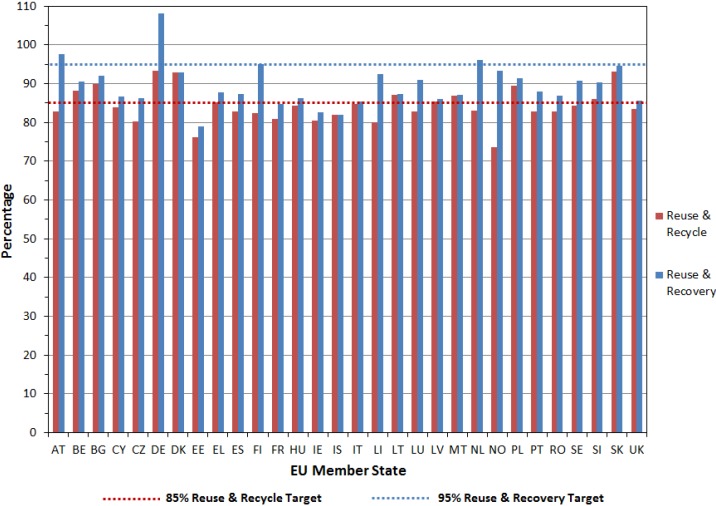
End-of-life vehicle (ELV) recovery and recycling rates reported by EU member states for 2011 [33].

Overcoming the obstacles surrounding the economics of recycling will likely require government involvement and initiatives. The EU ELV legislation, for example, requires 95% of a vehicle to be processed for “reuse and recovery” and 85% be processed for “reuse and recycling” by 1 January 2015 [11]. Vehicle manufacturers are economically responsible for the ultimate recyclability of vehicles, including the end-of-life treatments, recycling, and recovery operations [32]. ELV recovery and recycling rates reported by EU member states for 2011 (refer to Figure 3) varied from about 74% to 93% for reuse and recycling and 79% to 97% for reuse and recovery [33]. It should be noted that the 2011 figures are the most current figures available. Germany (*i.e.*, country code “DE”) reported reuse and recovery rates in excess of 100% which may imply that more materials were reused and recovered than generated. This may be possible if materials were imported for reuse and recovery. Based on the 2011 statistics, 63% of EU member states have yet to attain the 85% EU reuse and recycling target and 87% to attain the 95% EU reuse and recovery target.

In China ELVs are managed under the End-of-Life Vehicle Recycling Regulations (enacted in 2001), the Automotive Products Recycling Technology Policy (implemented in 2006) and the Regulation of Remanufacturing of Pilot Automotive Parts (enforced in 2008) [34]. Collectively they establish an ELV collection system that controls how vehicles may be used and managed at end-of-life. The system promotes improved safety by preventing the illegal refurbishment and use of old age vehicles, as well as controls how ELV-derived parts and materials may be reused. Similarly to EU’s ELV Directive, China’s ELV regulatory system, also sets ELV recycling targets, about 85% by 2010 (minimum 80% material recycling), about 90% by 2012 (minimum 80% material recycling) and about 95% by 2017 (minimum 85% material recycling) [34].

Japan’s Law for the Recycling of End-of-Life Vehicles (enforced in 2005) was established, in part, to address the problem of illegal dumping of automotive shredder residue (ASR) and diminishing space in disposal sites. The ELV Recycling Law sets recycling targets for airbags (85%) and ASR: 30% from 2005 to 2009, 50% from 2010 to 2014 and 70% from 2015 on. Automobile manufacturers and importers are responsible for the recycling of the ELVs. Unlike the EU ELV directive, Japan’s ELV Recycling Law does not stipulate recycling or recovery targets with respect to the total vehicle weight [34].

In Canada and the United States, regulation of ELV management facilities primarily focuses on business and operating practices as opposed to the regulation of the retired vehicles themselves [35]. The regulatory mechanisms (legal statutes, regulations, bylaws and voluntary mechanisms, such as best management practices (BMPs)) are applied to ELV management activities, such as, pollution prevention and control of air contaminant emissions, waste water discharges, and waster generation and disposal, as well as site use and materials storage practices [35]. The recycling of ELVs and ELV-derived parts and materials is principally a market drive system with used parts and scrap metal prices driving high recycling rates [36], which have been estimated to be as much as 86.3% by weight of ELVs processed in the [32]. Although there is no extended producer responsibility (EPR) or product stewardship legislation for ELVs managed in Canada and the United States, EPR-based initiatives have been launched for a variety of ELV-derived materials, or are under consideration (refer to Table 2).

**Table 2:** Examples of extended producer responsibility (EPR)-based initiatives for ELV-derived materials in Canada and the United States.

EPR-Based Initiative	Program	Jurisdiction	Source
Automotive mercury-containing switches	National Vehicle Mercury Switch Recovery Program (NVMSRP)	United States	[37]
	Switch Out Program	Canada	[38]
Used tires	Ontario Tire Stewardship (under Ontario Waste Diversion Act 2002)	Ontario	[39]
	Balanced Budget Act—Introduction of an environmental fee at time of new tire purchase	Quebec	[40]
Used oil and oil filters	Ontario Regulation 85/03, Used Oil Material (under Ontario Waste Diversion Act 2002)	Ontario	[41]
	Regulation respecting the recovery and reclamation of used oils, oil or fluid containers and used filters	Quebec	[40]
ELVs and ELV-derived materials	Proposed diversion of ELVs and ELV-derived materials from landfill (proposed changes under Ontario Waste Diversion Act 2002)	Ontario	[42]
	National ELV Environmental Management System (proposed)	Canada	[43]

Based on current markets and economics, any dismantling operation over and above what currently takes places will likely require a subsidy to compensate for the economic loss that would be incurred by the dismantler or recycler [43]. The introduction of incentives to push recycling may be one possible solution to promote segregation at the dismantling stage. Subsidies at the dismantling level may encourage separation of parts that are often not of significant value. For example, in the Netherlands, the dismantling, logistics, and sales chain for polyurethane foam is subsidised by Auto Recycling Netherland [43]. Subsidies could also be provided to drive research and development of the some of the technologies that are still not commercially available. Such subsidies may assist with “jump starting” the recycling process. Furthermore, introducing legislation which requires minimum recycling rates would drive the research and development of recycling technology and the development of partnerships. Legislation, such as the EU directive that 95% of vehicles disposed after 2015 must be recyclable will also help to drive research and development of technology and partnerships to achieve these milestones. Another possible avenue is to raise the cost (or “taxing”) production processes which rely completely on virgin materials while providing incentives to incorporate recycled materials.

Legislation could also help with creating a market for recyclates. For example, legislation requiring manufacturers to design new vehicles with a minimum percentage of materials obtained from SR would help to develop a real market for end of life vehicle materials. Investment in infrastructure should focus on one of two avenues: targeting more complete dismantling or developing SR processing technologies such as Polyfloat^®^ to achieve separation post-shredding. Interestingly, while the U.S. and Canada have largely resisted EU style regulations on recycling in the automotive industry, the EU regulations have spurred global, often voluntary actions related to end-of-life issues among a number of the automotive OEMs due to the interconnected and global nature of the automotive sector.

#### 3.2.4. Knowledge Transfer (Move to Uniform Mix of Materials)

A previous case study has demonstrated that a knowledge gap exists between what manufacturers believe are positive environmental choices and end of life reality [22]. Additionally, there is the obstacle of conveying information, such as the location of recyclable materials, from the manufacturer to the ELV dismantler. These gaps need to be addressed through collaborative efforts. One tool that has been developed is the International Dismantling Information System (IDIS) database [44]. The IDIS database provides information to assist with vehicle dismantling; specifically, it shows where materials can be found within the vehicle. This database has the potential to increase recyclability by assisting dismantlers with identifying where recyclable plastics are within the vehicle; however, there are still several limitations such as reporting inconsistencies between manufacturers.

Manufacturers also need to be aware of the limitations surrounding recycling of thermoset materials and specify these only in situations where their properties are required. Awareness of the end of life routes for each material they consider as well as of the contradiction between heterogeneity and recyclability is also important. Rather than consider whether a material is recyclable, it is more important to consider the likely end of life fate. Design for Resource Efficiency (DfRE) is a concept in which the end of life scenario is included within the design phase with regards to how the resources can be extracted from the dismantling and recycling processes [45]. This gap can be bridged through publications and conferences and through forging partnerships between manufacturers and researchers. One important factor to convey to manufacturers might be how reducing the diversity of materials during production may increase recyclability.

### 3.3. Alternative 3—Pursue Recovery without Plastics Segregation (Energy Recovery)

Recovering unsegregated ELV materials (including plastics) is commonplace in the metals shredding industry. Since shredding was first introduced in the early 1960s [46], the basic materials recovery technologies have matured and are in wide use. End-of-life vehicle (ELV) hulks, end-of-life large appliances (ELLAs), and construction, renovation and demolition (CRD) waste are typical shredder feed stocks. These are comminuted through large metal shredders (hammermill, typical) producing a heterogeneous mixture of metals (ferrous and non-ferrous) and non-metals (e.g., plastics, glass, textiles, rubber/elastomers, paper, wood, ceramics, *etc.*) [20]. From this shredded mixture, magnetic ferrous metals (cast iron, carbon steel, low grade stainless steels) are recovered using magnetic separation systems (e.g., magnetic drum, magnetic head pulley, magnetic belt separators, *etc.*) [47]. Non-magnetic, non-ferrous metals (aluminum, copper, zinc, nickel, high-grade stainless steels, lead, *etc.*) are recovered and concentrated principally using eddy current rotor separators, commonly in combination with screening devices such as trommel or vibrating deck screens to remove fines [20,48,49]. The low density, non-metallic materials are removed from the heavier, metal-rich materials using air suction and conveyed to air elutriation devices for recovery (e.g., vertical air classifiers, such as Z-box separators and air cyclone separators) [20].

The “left overs” of this process is a heterogeneous mixture of non-metallic materials with a small proportion of non-recoverable metals, and is referred to as shredder residue (SR) or shredder fluff. It is also commonly referred to as auto shredder residue, which can be misleading given that it is typically generated as a result of shredding ELVs along with ELLAs and CRD waste. SR is disposed of in landfills [20], but can be costly, particularly if it is deemed hazardous due to the presence of sufficient quantities of leachable contaminants, such as mercury (from mercury switches), polychlorinated biphenyl or PCB (from PCB components in ELLAs or CRD waste commingled with ELVs), or lead (from soldered wire connections) [20]. Alternative SR management mechanisms have been explored and include reuse, recycling and energy recovery options, such as:

- reuse of SR as landfill day cover [50,51,52];
- reuse of SR as a hydroponic garden growing medium [53];
- use of the organic portion of SR as an alternative fuel source or reducing agent in blast furnaces [50,54,55];
- use of SR as an alternative fuel and mineral feedstock for cement production [55,56];
- recycling of SR in the manufacture of composite plastic building products, e.g., plastic lumber [57,58];
- pyrolysis of SR to produce a synthetic coal/fuel product [51,55,59,60,61,62]; and
- recycling of SR plastics, involving the conversion of the plastics into low molecular weight hydrocarbons (such as via low-temperature, catalytic conversion) for reuse as chemicals or fuels [55,62,63].

The aforementioned reuse and recycling mechanisms for non-segregated SR materials have been generally limited to proposed, experimental or conditional applications. Although the above alternatives may be viable and seen as environmentally beneficial ways of reusing or recycling SR, are they sustainable?

Using SR as landfill day cover is practiced in the U.S. and Canada, but landfill facilities require prior approval to use SR as alternative day cover (ADC). Even as ADC, SR takes up landfill space and concerns about SR quality may arise, due to potential contaminants, for example, automotive fluids, PCBs, or leachable heavy metals, unless SR quality is regularly monitored [51,52].

Energy and resources are necessary both to shred and recover the materials. Pretreating the SR materials may be required (e.g., screening, air elutriation, froth flotation, *etc.*) to upgrade and concentrate the organic portion of SR (principally plastics) prior to it being used in energy recovery applications or recycled into manufactured plastic products [64,65,66]. The use of SR organic materials for energy recovery results in the consumption of non-renewable resources in secondary processes. If the amount of energy used to generate the SR and transform it into a useable fuel is greater than the calorific value of the SR-derived fuel, justifying SR as a fuel source may be difficult.

### 3.4. Alternative 4—Move to Renewable Plastics

Social and economic progress of the 21st century has propelled renewable plastics to the forefront of the plastics industry. Bio-plastics are now considered a viable alternative to conventional petroleum based plastics. Bio-plastics, even as an emerging industry, has many virtues worthy of the transition away from petroleum based plastics in the current literature [67,68]: the two most influential factors are renewability and biodegradability.

Renewability with relation to bio-plastics can be defined as plastics manufactured from sources that are replenished naturally on an anthropological time frame. Some examples of renewable sources of bio-plastics are corn, sugar cane and algae. In the short and medium term, the price fluctuation due to the finite supply of fossil oil can be minimized by utilizing renewable biomass as raw materials [69]. In the long term, the development of bio-plastic is driven by the acknowledged, definite supply of fossil oil [70]. By making the transition towards utilizing bio-plastics, social concerns, production cost and environmental impacts can be lowered.

Biodegradability is commonly used to define a product’s ability to completely disintegrate chemically and biologically; however, there are many kinds of biodegradability not well known among the general public. For bio-plastics, degradability under commercial composting conditions and household composting conditions are expected. EN13432 and ASTM D6400 are the two standards that define the industrial composting [71], while household composting conditions are not standardized. Similar to renewability, the biodegradability of bio-plastics reduces the environmental impacts of plastic products and moves the plastic industry overall to a greater level of long-term sustainability.

For automotive applications, bio-plastics are currently limited to interior trims and non-structural components with ongoing research to improve the material properties for universal application. The mature production infrastructures of petroleum plastics, however, remain obstacles to bio-plastics becoming main stream commodity plastics. Future social pressures and subsequent market trends and even government legislation will dictate the eventual route of bio-plastics progression.

### 3.5. Alternative 5—Move away from Plastics

When plastics were first introduced into the automotive industry, their light weight and mouldability were seen as improvements over metals for certain applications. However, while plastics may reduce the overall weight of a vehicle, they are not readily recoverable and recyclable compared with metals [34]. The initially perceived benefit to switching to plastics is being questioned. A comprehensive life cycle analysis might expose metals as having a smaller environmental impact associated with their tertiary life cycles compared to the end-of-life scenario for currently unrecoverable plastics [72].

Whether it would be beneficial to reduce plastics use and re-emphasize metals use in automotive applications depends on a variety of factors. Ferrous metals are readily recyclable from ELVs using magnetic separators, while plastics add to the complexity within the shredding residue [34]. While dismantling ELVs can further reduce the environmental impacts, not all plastics can be dismantled from the vehicle [72]. Ironically, using metal clips and screws instead of typical glues to join plastics together may improve separation during recovery [73]. Nevertheless, metals are generally heavier than plastics, and results in greater impacts during the use-phase from an LCA perspective. Further investigations from an LCA perspective contrasting the use-phase and end-of-life phase from metals compared with plastics could identify whether moving away from plastics would be a worthwhile alternative [74].

## 4. Recommendations and Conclusions

Five alternatives were discussed in relation to the options for plastics and composites from ELVs. One of the first, critical questions is whether or not conventional plastics are the best choice for the application being considered. In some cases, bio-plastics or metals may provide superior functionality and environmental performance over the life cycle of the vehicle given the challenges of recovering plastics. The mindset that plastics are a preferred material choice needs to be challenged.

In cases where conventional plastics are used, the best recovery alternative is likely a combination of the reviewed options; for example, combining recycling and energy recovery steps. Segregating the larger, cleaner material pieces for recycling and sending the remainder for energy recovery is a common choice; however, future efforts should look to increase the proportion destined for recycling. For thermoset materials, emerging techniques are showing promising results towards the development of new materials that can have the desirable properties of thermoset plastics while being recyclable at the ELV stage. Several of the alternatives could exist in combination to comprehensively address the challenges of recycling with the overall goal of improving the economics and efficiency of identifying, separating, and recovering plastics.

In the absence of major investment in infrastructure and new technology, an alternative may be to promote increased uses of SR. Post-shredding SR treatment technologies can play an important role in increasing overall recycling rates. Although these technologies exist, they are currently available on a limited commercial scale [66]. Technologies are available; however, it will take incentives and legislation to commercialize them. Legislation could advance this initiative by requiring manufacturers to incorporate SR materials into new design.

Subsidies and legislation may play a critical role in addressing the abovementioned obstacles. A major limitation of sustainable recycling of these products is cost. Incentives could propel some of these alternatives forward. Providing penalties for waste generators and credits for recyclers and material recycling initiatives is one option, while subsidies could be provided at various levels.

Incorporating recovery principles into the product design stage is critical. Firstly, informing manufacturers of the likely EOL fate of their materials is an important component. Also, the need for manufacturers to adapt the mindset of sustainable design is imperative. This can be achieved by reducing the number of types of materials that are used in order to streamline segregation activities, or alternatively, selecting materials with easy-to-recover properties in applications where plastics recovery will be difficult.

Establishing applications and sound markets for recyclates through collaborative efforts and partnerships will be key to closing the materials reuse loop. Where energy recovery is the only option, waste to energy practices can be implemented.

In conclusion, plastics play an important role in the automotive industry, but there are obstacles to overcome in order to ensure that they are sustainable. Pursuing recycling in the automotive industry will require improvements in the quality of the recyclate, establishing industry partnerships, incentives and legislation, and knowledge transfer to industry stakeholders. Implementing some of the innovative alternative SR management techniques will require significant, further research to determine if they are sustainable practices. Moving to renewable plastics will be determined by industry maturity and progression. Switching away from plastics and back to metals will require proper analysis between the impacts from the use-phase and end-of-life phase from an LCA perspective. Several options exist for the EOL fate of plastics but none truly stand out as the preferred alternative: future research is critical to determining what the ideal combination of alternatives might be for long term sustainability. Finally, many of the issues raised and lessons learned to date from the use of plastics in vehicles may be applicable to the use of plastics in other increasingly complex, durable consumer and industrial items.
